# EWSR1 rearrangement in papillary thyroid microcarcinoma is related to classic morphology and the presence of small-cell phenotype

**DOI:** 10.17305/bjbms.2021.6181

**Published:** 2021-08-09

**Authors:** Bozidar Kovacevic, Ana Caramelo, Vesna Skuletic, Snezana Cerovic, Catarina Eloy

**Affiliations:** 1 Department of Pathology, Institute of Pathology and Forensic Medicine, Military Medical Academy, Belgrade, Serbia; 2 Ipatimup Diagnostics, Institute of Molecular Pathology and Immunology of Porto University, Ipatimup, Porto, Portugal; 3 Department of Pathology Medical Faculty, University of Porto, Porto, Portugal

**Keywords:** Papillary thyroid carcinoma, small cells, Ewing sarcoma breakpoint region 1, *EWSR-FLI1*, carcinoma of the thyroid, Ewing family tumor elements

## Abstract

The Ewing sarcoma breakpoint region 1 (*EWSR1*) rearrangements with unknown genes were detected in a high percentage of classic variants of papillary thyroid carcinoma. The small-cell carcinoma of the thyroid with Ewing family tumor elements typically presents with *EWSR1-FLI1* rearrangement suggesting the possible role of *EWSR-FLI1* translocation in the loss of thyroid differentiation and acquisition of a small-cell phenotype. To determine the frequency and association of *EWSR1* rearrangements, particularly the *EWSR1-FLI1* fusion with clinicopathological features of papillary thyroid microcarcinoma (m-PTC) and the presence of small cells, we analyzed a series of 99 m-PTCs using the fluorescence *in situ* hybridization method. Ninety cases (90.9%) of m-PTC were positive for small cells. This group of m-PTC has shown more often invasive growth, lymphatics invasion, and moderate/extended intratumoral fibrosis. Three cases out of 99 were inconclusive for *EWSR1* rearrangement. Eighty-nine (92.7%) and 27 (28.1%) out of 96 m-PTC cases were positive for *EWSR1* rearrangement and *EWSR1-FLI1* fusion, respectively. m-PTC with classical architectural pattern presented more frequently with *EWSR1* rearrangement relative to m-PTC with other patterns (*p* = 0.005). Other clinicopathological features were not related to the presence of *EWSR1* rearrangement or *EWSR1-FLI1* fusion. The percentage of small cells present significantly correlated with the percentage of cells positive for *EWSR1-FLI1* fusion (*p* = 0.05) and *EWSR1* rearrangement (*p* < 0.001). *EWSR1-FLI1* fusion is not rare in m-PTC and it is associated with the acquisition of small-cell phenotype. The *EWSR1* gene rearrangement is a frequent event in m-PTC and is related to the classical pattern of m-PTC.

## INTRODUCTION

The Ewing sarcoma breakpoint region 1 (*EWSR1*) gene encodes a protein that is involved in various cellular processes, including regulation of gene expression at the level of transcription initiation, signal transduction, and mRNA transport [1,2]. In addition to its role in gene expression, *EWSR1* is also a player in preserving genome integrity and cellular senescence, as previously demonstrated [1,2]. Rearrangements between *EWSR1* and other genes lead to the formation of hybrid proteins with oncogenic potential associated with neoplastic cell ­transformation, both in the mesenchymal neoplasms and in other cancer models [3,4]. *EWSR1* rearrangements with different partner genes are related to different phenotypes of neoplasms, namely small-cell, spindle-cell, clear-cell phenotype or neoplasms with distinctive myxoid stroma [3]. The Ewing Sarcoma family of tumors (ESFT) encompasses tumors with common small-cell phenotype and molecular features that include balanced reciprocal translocations between *EWSR1* and genes of the *ETS* family of transcription factors. The two most common rearrangements include fusion of *EWSR1* with *FLI1* and *ERG* genes, which were described in 85% and 10% of cases, respectively [3,4]. *EWSR1* rearrangement is also present in a small number of non-mesenchymal tumors including benign and malignant myoepithelial tumors and hyalinizing clear cell carcinomas of the salivary glands [5-7], hidradenoma of the skin [8], clear cell odontogenic carcinoma [9] and primary hyalinizing clear cell carcinoma of the thymus [10]. Rare cases of *EWSR1* translocation were also described in renal cell carcinoma and colon adenocarcinoma [11,12].

In the group of thyroid tumors, the small-cell carcinoma of the thyroid with Ewing family tumor elements (CEFTE) typically discloses *EWSR1-FLI1* rearrangement [13-15]. This rearrangement was also described in rare cases of papillary thyroid carcinoma (PTC), suggesting that PTC may be a ­possible origin of CEFTE [13-15]. The same authors question the possible role of *EWSR-FLI1* rearrangement in the loss of thyroid differentiation and acquisition of a small-cell phenotype. In PTC, the *EWSR1* rearrangements with unknown genes were also detected in a high percentage of classic variants of PTC cases [13].

Papillary thyroid microcarcinoma (m-PTC) is a PTC with 10 mm or less in the highest dimension [16]. m-PTC is the most common variant of PTC and discloses an excellent prognosis. Despite its favorable general clinical behavior, a group of m-PTC includes tumors with a potentially aggressive course, such as those giving rise to loco-regional metastases, recurrence of the disease, rarely, and distant metastasis. m-PTC can disclose varied growth patterns and cellular features, as described in larger PTC [17-19]. The mitogen-activated protein kinase pathway is the most frequently altered signaling pathway in m-PTCs, being the presence of *BRAF* V600E mutation related to a distinctive phenotype (classic morphology, tall cell features, infiltrative borders, presence of stromal fibrosis and psammoma bodies, and well-developed nuclear features typical of PTC). The association of *BRAF* V600E mutation and aggressiveness of m-PTC is questionable [17,19,20]. Besides the *BRAF* V600E mutation, mutation of the *RAS* gene, like in larger PTC is related to the follicular pattern of m-PTC, as well as, *RET/PTC* rearrangements are associated with the classical pattern and/or the so-called “Bonsai phenotype” of m-PTC [16,21].

This study aimed to evaluate the frequency of *EWSR1* rearrangements, particularly the *EWSR1-FLI1* fusions, in m-PTC, their possible relation with clinicopathological features of m-PTC and their possible association with the presence of a small-cell phenotype.

## MATERIALS AND METHODS

### Patient selection

A series of 99 m-PTC cases with a minimum size of 5 mm was selected from the archive of the Institute of Pathology and Forensic Medicine of Medical Military Academy (Belgrade, Serbia) according to the previously described methodology [22].

### Clinicopathological features of m-PTC

General clinical information such as age, gender, and type of surgical procedure were obtained from the medical records of the patients. The following pathohistological parameters were reviewed for each case according to the previously described methodology [22]: Tumor size, presence of LN metastases, presence of distant metastases, extrathyroid extension, predominant architectural pattern of the tumor, growth pattern, multifocality, lymphatics invasion, vascular invasion, intratumoral fibrosis, stromal calcification, and psammoma bodies.

**FIGURE 1:**
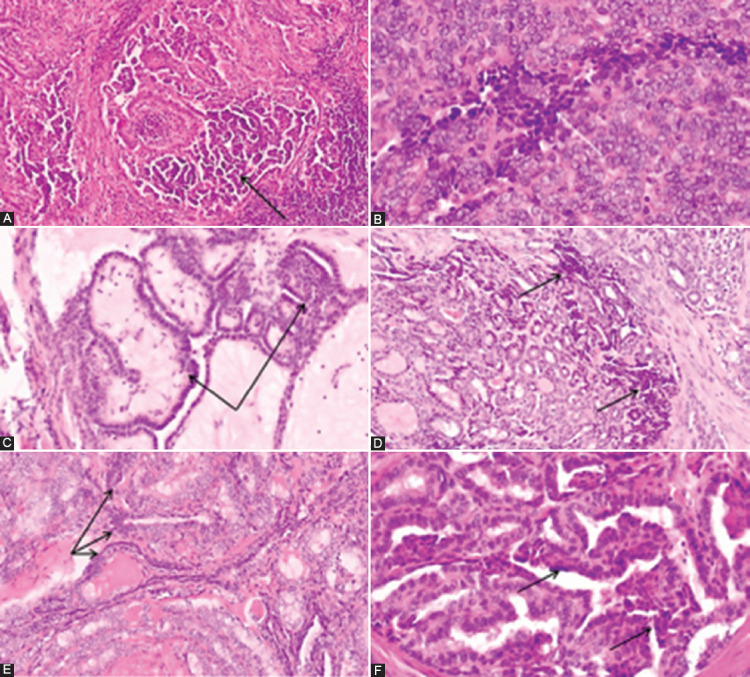
The morphology of small cells in papillary thyroid microcarcinoma. (A) Short papillary structures and cellular tufts composed of small cells with complete or incomplete nuclear features of PTC and occasionally with hyperchromatic appearance (arrow); (B) Small cells with hyperchromatic nuclei in a solid pattern m-PTC* (arrow); (C) Tumor area composed by packed edematous papillary structures covered with small, cuboidal cells with hyperchromatic nuclei (arrow). (D and E) Small cells with hyperchromatic nuclei (arrow), mixed with cells of typical PTC nuclear features; (F) Small cells at the top of the neoplastic papillae (arrow); (A-F) details from slides scanned at ×400; **H&E. *Papillary thyroid microcarcinoma. **Hematoxylin and eosin.

The presence of small-cell phenotype was also analyzed through the presence of small cells. The number of small cells was determined semi-quantitatively after analysis of the whole sections of the tumor and it was estimated as a percentage of the total number of tumor cells. Small cells were defined as cells smaller than surrounded tumor cells of PTC, not larger than 4x lymphocyte size, with scant or inconspicuous cytoplasm, hyperchromatic oval or irregular nuclei or nuclei with incomplete features of PTC (moderate nuclear enlargement with chromatin clearing, with or without nuclear grooves or pseudoinclusions). The cellular clusters which appear as small nests of highly hyperchromatic, deformed nuclei usually present at the edge of peripherally localized m-PTC near to surgical resection margins, were considered as an artifact and were not counted as small cells [23]. Slightly distorted cells present in the deeper parts of the tumor surrounded by well-preserved small cells and regular cells of m-PTC were considered as small cells, as small cells commonly undergo crushing artifact [24]. The cases of m-PTC with less than 1% of small cells were considered as negative for small cells, and cases with at least 1% of small cells were counted as m-PTC positive for small cells. Figure 1 is shown the morphology of small cells in m-PTC. Two pathologists (BK, SC) reviewed the Hematoxylin and eosin-stained (H&E) slides of all cases and histological parameters were analyzed according to the classification of the World Health Organization Classification of Tumors of Endocrine Organs [16]. The staging of disease was performed according to the AJCC/TNM Staging System for PTC (8^th^ Edition) [25].

### Fluorescence *in situ* hybridization (FISH) testing

Tissue microarrays (TMA) from the 99 cases were built and an H&E stained section from each TMA paraffin block were produced to evaluate the representativity of the m-PTCs in each core. FISH was performed according to the methodology previously described for the technique and for the establishment of the cutoff [13]. FISH technique was performed in 4mm tissue sections obtained from the TMAs paraffin blocks. The sections were extended on polysine slides, following deparaffinization with xylene and alcohol. Antigen retrieval was performed using citrate buffer pH6.0 at 95°C (Thermo Scientific, Waltham, MA USA) and enzymatic digestion with pepsin at 37°C (Sigma-Aldrich Quimica, Sintra, Portugal). Afterward, co-denaturation at 79°C and hybridization at 37°C were performed overnight in a SPOT-Light CISH Hybridizer with a ZytoLight SPEC *EWSR1-FLI1* TrickCheck Probe (ZytoVision, Bremerhaven, Germany). Nuclei were counterstained with DAPI (ZytoVision, Bremerhaven, Germany). Adequate positive and negative controls were used in each set of slides. Fluorescence hybridization signals were analyzed and recorded with a D-Sight Fluo 2.0 (Menarini, Florence, Italy).

In each case, 50 nuclei were evaluated, and records were undertaken regarding *EWSR1* rearrangement, *EWSR1* monosomy, *EWSR1* polysomy, and *EWSR1-FLI1* fusion. Cases with least than 50 nuclei with preserved signals due to technical reasons were classified as inconclusive. *EWSR1* rearrangement was contemplated if a signal separation of the 5’-orange and the 3’-green of the *EWSR1* probe was present. If this signal separation was simultaneous with the fusion of the 5’-orange signal of *EWSR1* and the blue signal of *FLI1*, the case was counted positive for *EWSR1-FLI1* fusion - t(11;22). A normal nucleus was accepted when exhibiting 2 signals fusion of *EWSR1 (*5’-orange and 3’-green) and 2 blue *FLI1* signals separated.

The cases were considered positive for *EWSR1-FLI1* fusion if this fusion signal was present in ≥2.4% of neoplastic nuclei. Positive cases for *EWSR1* rearrangement were considered in cases when break-apart signal was present in ≥2.4% of neoplastic nuclei. Polysomy was acknowledged when the number of signals per neoplastic nucleus was superior to those observed in the normal nuclei. Monosomy was acknowledged when the number of signals in each neoplastic nucleus was inferior to those observed in the normal nuclei.

### The relationship between nuclear size and *EWSR1* rearrangement

Of the five selected cases of m-PTC with the highest ­percentage of nuclei with *EWSR1-FLI1* fusion and *EWSR1* rearrangement, the perimeter of the nucleus of cells was measured using the morphometric tools of the D-Sight Navigator viewer. The perimeter of the neoplastic nuclei was compared after its division into three groups: Group 1- neoplastic nuclei without *EWSR1* rearrangement; Group 2 - neoplastic nuclei with *EWSR1* rearrangement, and Group 3 - neoplastic nuclei with *EWSR1-FLI1* fusion.

### Ethical statement

This work was performed in archived paraffin tissue that remained from the diagnosis of the thyroid samples of patients that were anonymized. The materials used for the work are not needed for diagnosis in the present nor in the future. The Ethics Committee of the Military Medical Academy and University of Defense, Serbia, approved this study (Number: 16-71; 5/29/2019).

### Statistical analysis

Statistical analysis of data was done with the statistical software package, SPSS Statistics 18. Most of the variables were presented as frequency of certain categories, while statistical significance of differences was tested with the Chi-square test (F test in case of expected frequencies <5). In case of continuous data, variables were presented as mean value ± standard deviation, minimal and maximal values. The Kolmogorov–Smirnov test was applied to assess the normality of distribution of continuous data. According to the results of this test, statistical significance between groups was tested by ANOVA test (*post hoc* Tukey test). Spearman correlation analysis was used to establish the relationship between parameters. All the analyses were estimated at minimal *p* < 0.05 level of statistical significance.

## RESULTS

In the selected cohort of 99 m-PTCs, the patients had a mean age of 49 ± 14.4 years, ranging from 19 to 85 years, at the time of diagnosis. There were 22 males (22.2%) and 77 females (77.8%). The mean tumor size was 7.6 ± 1.5 mm. Cervical LNs metastases were present in 30 cases (30.3%). Fifteen patients had LNs metastases in the central neck compartment and 15 patients had lateral cervical LNs metastases. None of the patients had documented distant metastases. In the database of pathohistological reports, six cases (6.06%) had histological confirmation of disease recurrence in regional LNs. According to the medical records, these patients received radioiodine therapy 6-8 weeks after thyroidectomy was performed. Most of the patients (*n* = 92; 92.9%) were Stage I and 7 of patients (7.07%) were Stage II of disease. Only three cases (3%) showed gross ETE. The classical architectural pattern of the tumor was the most common and was present in 55 cases (55.6%), followed the follicular pattern in 38 cases (38.4%) and solid in six cases (6.1%). The infiltrative growth pattern was observed in 72 cases (72.7%) and 27 cases (27.3%) showed an expansive/circumscribed growth pattern. Sixty-four cases (64.6%) disclosed lymphatics invasion and vascular invasion was present in 15 cases (15.2%). Tumor multifocality was observed in 81 cases (81.81%). Moderate/extended intratumoral fibrosis was observed in 57 cases (59.4%), and absent/mild intratumoral fibrosis was observed in 39 cases (40.6%). Stromal calcification was observed in 33 cases (33.3%) and psammoma bodies were observed in 46 cases (46.5%).

In the studied cases, the mean percentage of small cells was 12.3% ± 16.9%, ranging from 0% to 80%. Nine cases (9.1%) of m-PTC were considered negative for small cells and 90 cases (90.9%) of m-PTC were positive for small cells. The mean percentage of small cells in the small cells positive m-PTC cases was (13.6% ± 17.2%) ranging from 1% to 80%. In the group of m-PTC positive for small cells the presence of invasive growth, lymphatic invasion, and moderate/extended intratumoral fibrosis were statistically more common than in the group of m-PTC negative for small cells. In regard to intratumoral occurrence of small cells, it was not possible to establish the specificity of their topographic distribution. Most commonly, small cells were present as single or clusters of cells mixed or surrounded by cells with typical PTC nuclear features. It was possible to notice areas of small cells in the vicinity, within but also away from intratumoral fibrosis. The clinicopathological features of m-PTC with and without the presence of small cells are described in Table 1.

**TABLE 1:**
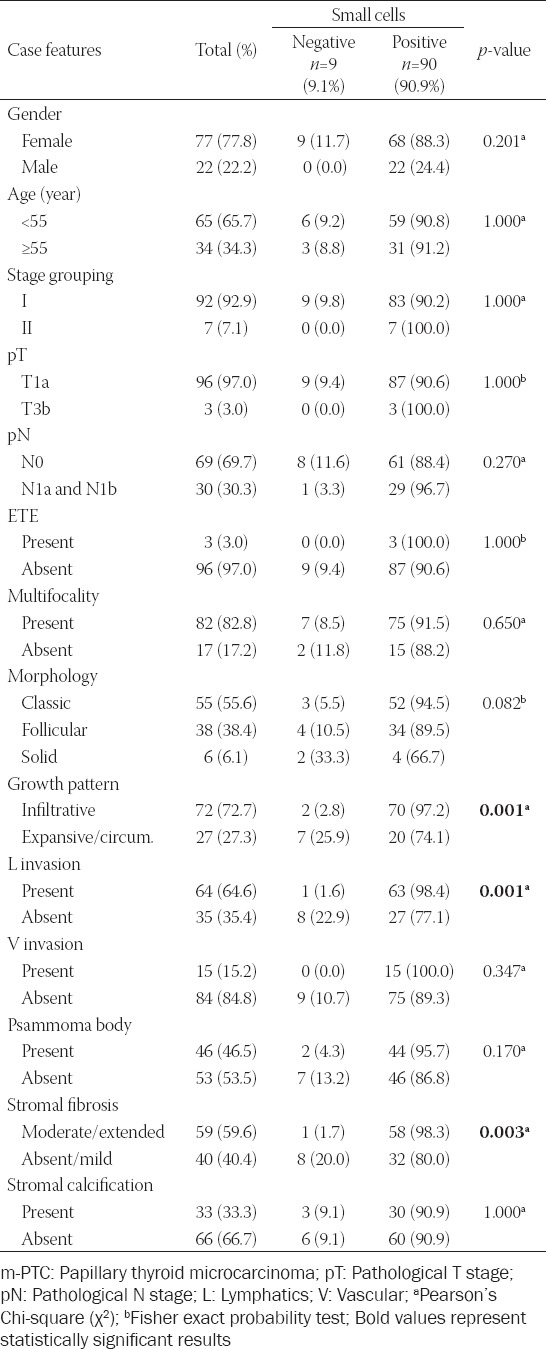
Clinicopathological features of m-PTC with small cells versus m-PTC without small cells

After FISH analysis, three cases (3.03%) without signals in less than 50 nuclei were considered inconclusive and excluded from further analysis. Inconclusive cases were in two females and one male patient and all cases were in group staging I. Two cases were classical and one was solid in morphology. Two of them had LNs metastases.

**FIGURE 2:**
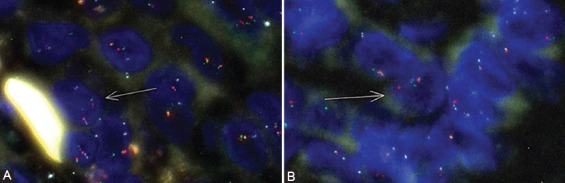
The FISH analysis of cases with *EWSR1* rearrangement and *EWSR1-FLI1* fusion. (A) The arrow shows a cell with a break-apart of *EWSR1*. This has one normal signal (5’ orange and 3’green) and two separated 5’ orange and 3’green signals of *EWSR1*, FISH* ×200; (B) The arrow represents one cell with the presence of the fusion of *EWSR1* with *FLI1*. This cell present one normal signal of *EWSR1* (orange and green signal’s) and one fusion with *FLI1* (orange and aqua signal), FISH* ×200. *Fluorescence *in situ* hybridization.

Eighty-nine out of 96 m-PTC cases (92.7%) were considered positive for *EWSR1* rearrangement. The mean percentage of cells with *EWSR1* rearrangement in the *EWSR1* positive m-PTC cases was (16.7% ± 9.7%) ranging from 4% to 66%. Figure 2 displays FISH analysis of cases with *EWSR1* rearrangement and *EWSR1-FLI1* fusion.

Twenty-seven cases (28.1%) were positive for *EWSR1-FLI1* fusion while 69 cases (71.9%) were negative. The mean percentage of cells with *EWSR1-FLI1* fusion in the *EWSR1-FLI1* fusion-positive m-PTC cases was (6.2% ± 2.6%) ranging from 4% to 12%.

**FIGURE 3:**
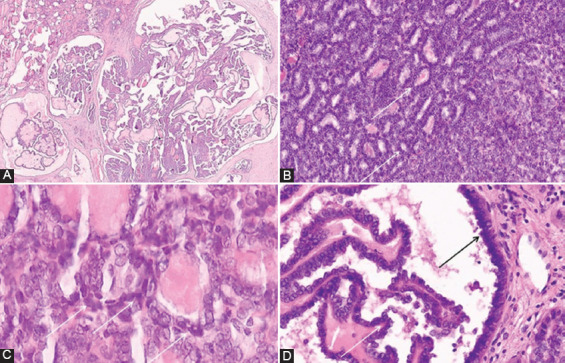
The morphology *EWSR1-FLI1* fusion positive and *EWSR1* rearrangement positive cases of papillary thyroid microcarcinomas. (A) Classic pattern m-PTC* with invasive growth and predominance of small cells; (B) m-PTC with the predominance of small cells arranged in the microfollicular pattern; (C) small cells cover papillary structures and cystic space (arrow) in *EWSR1- FLY1* positive m-PTC; (D) small cells surround follicles (arrow) with typical nuclear features of PTC in *EWSR1-FLY1* positive case; (A-D) Details from slides scanned at ×400; **H&E. *Papillary thyroid microcarcinoma. **Hematoxylin and eosin.

Regarding the occurrence of small cells in m-PTC, the percentage of their presence significantly correlated with the percentage of cells with *EWSR1-FLI1* fusion (ρ = 0.201; *p* = 0.05) and *EWSR1* rearrangement (ρ = 0.349; *p* <0.001), and it was in a negative correlation with the percentage of neoplastic nuclei with monosomy (ρ = -0.202; *p* = 0.048). This association is further supported by the results of five m-PTC cases that have shown the statistically significant smaller size of neoplastic nuclei with *EWSR1-FLI1* fusion and *EWSR1* rearrangement in comparison to the size of neoplastic nuclei of m-PTC without *EWSR1* rearrangement. Neoplastic nuclei with *EWSR1-FLI1* fusion were also smaller than nuclei with *EWSR1* rearrangement, but statistically, a significant difference was proven only in case number 5 (*F* = 5.889; *p* = 0.006; Tukey *post hoc* test; *p* = 0.047). Table 2 summarizes the results of the study of the nuclear perimeter of individual m-PTC cases and the total number of analyzed nuclei. Figure 3 documents morphology of *EWSR1-FLI1* fusion positive and *EWSR1* rearrangement positive m-PTC cases with the highest percentage of small cells, as well as morphology of small cells of *EWSR1-FLI1* positive m-PTC.

**TABLE 2:**
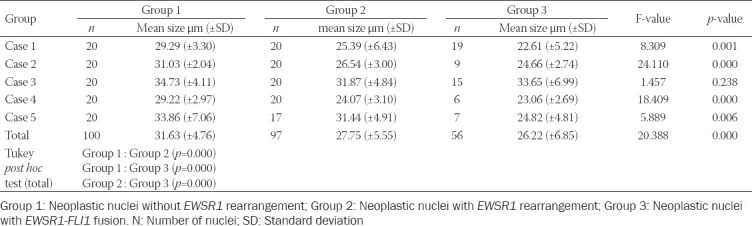
Comparison of papillary thyroid microcarcinoma nuclear size in selected cases with or without *EWSR1-FLI1* fusion and *EWSR1* rearrangement

The percentage of monosomic neoplastic nuclei in m-PTC ranged from 0% to 72% (mean 13.7% ± 12.7%) and was detected in 88 cases (91.7%). Eight cases were negative for monosomy and in six cases monosomy was present in 2.0% of neoplastic nuclei. An increasing percentage of neoplastic nuclei with monosomy of *EWSR1* gene in more than 2.4% of neoplastic cells is in a statistically significant negative correlation with the percentage of *EWSR1* rearrangement (ρ = –0.536; *p* < 0.001), the presence of LN metastasis (ρ = –0.319; *p* < 0.002) and lymphatics invasion (ρ = -0.252; *p* < 0.013).

Only one (1.0%) out of 96 cases disclosed the presence of polysomy of *EWSR1* gene that was detected in 2% of nuclei.

*EWSR1* rearrangement was more frequently observed in m-PTC with classical than in m-PTCs with follicular or solid patterns (100.0% vs. 84.2% and 80.0%, respectively; *p* = 0.05). An increasing percentage of *EWSR1* rearrangement within a group of *EWSR1* positive m-PTC statistically significantly correlated only with the presence of lymphatic invasion (ρ = 0.204; *p* < 0.046). Tables 3 and 4 summarize the clinicopathological features of m-PTCs with and without *EWSR1* rearrangement and *EWSR1-FLI1* fusion.

**TABLE 3:**
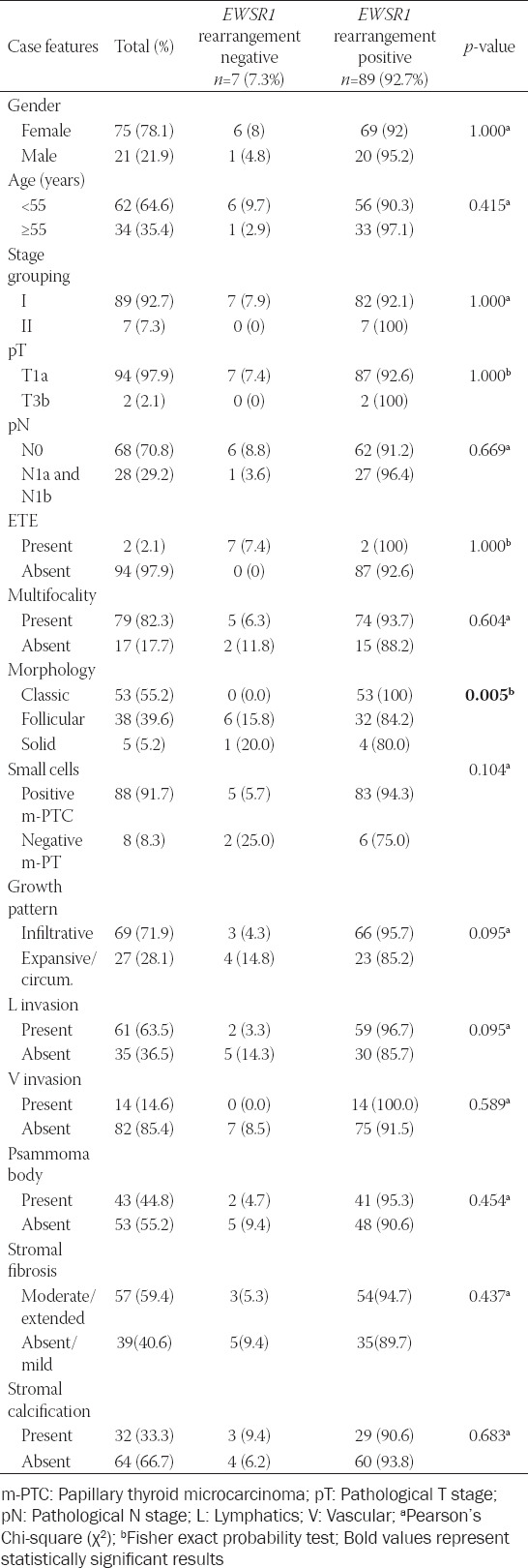
Clinicopathological features of m-PTC with *EWSR1* rearrangements versus m-PTC without *EWSR1* rearrangements

**TABLE 4:**
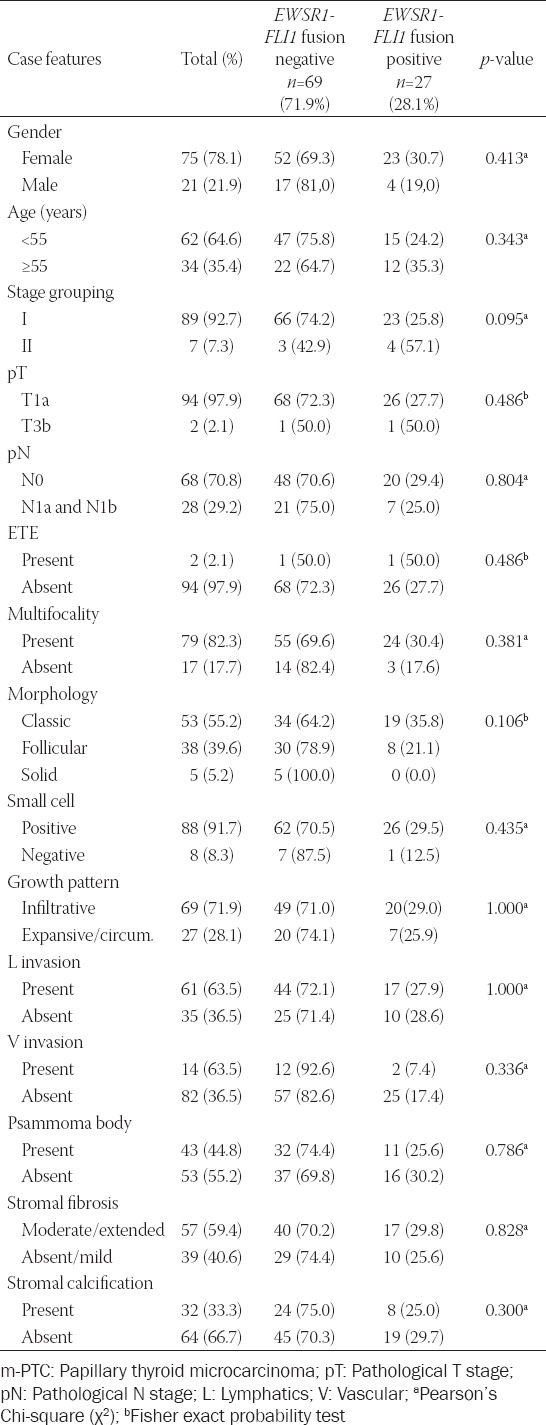
Clinicopathological features of m-PTC with *EWSR1*-*FLI1* fusion versus m-PTC without *EWSR1*-*FLI1* fusion

The percentage of cells with *EWSR1*-*FLI1* fusion in m-PTC does not correlate with the presence of *EWSR1* rearrangement or gene monosomy and polysomy.

## DISCUSSION

*EWSR1* is one of the most frequently altered genes in PTC [13]. The meaning of *EWSR1* rearrangement is unknown, but *per se*, looks like it does not have any significant clinical meaning, as documented previously and in the present study, namely, by the lack of association between *EWSR1* rearrangement and clinicopathological features such as the presence of LN metastasis and distant metastasis. This finding, regarding the high frequency of *EWSR1* rearrangement in m-PTC, is in line with available evidence that there is no single genetic change that alone can predict the aggressiveness of m-PTC [17,19].

*EWSR1* rearrangement represents one of the most frequent molecular events in mesenchymal neoplasm [3,4], and a rare genetic event, that has an increasing number of reported cases, in non-mesenchymal tumors [5-13]. In the present study, the rearrangement of the *EWSR1* gene was detected in all cases of m-PTC with a classical pattern. The frequency of *EWSR1* gene rearrangements in m-PTC with other patterns was significantly lower even absent as occurred in solid pattern m-PTCs supporting the phenotype-related significance of *EWSR1* rearrangements. Previously, it has been demonstrated that the classic architectural pattern of m-PTC and the classic variant of PTC were also related to the presence of *BRAF V600E* mutation and *RET-PTC* rearrangement. As we did not analyze other molecular alterations and their ­association with *EWSR1* rearrangement, we cannot exclude that different combinations of genetic alterations in association with *EWSR1* rearrangement are responsible for the development of the classical pattern m-PTC.

Our results have been shown that the region of *EWSR1* is highly prone to frequent alteration in m-PTC, not only as recurrent balanced aberrations but also as genes monosomy. The high frequency of *EWSR1* monosomy has not been shown to be associated with clinicopathological features of m-PTC. Moreover, m-PTC cases without *EWSR1* monosomy were those showing LNs metastases and lymphatics invasion than m-PTC with *EWSR1* monosomy. This fact can suggest that the loss of the *EWSR1* gene from neoplastic cells of m-PTC can be perceived as a secondary aberration that accumulates during tumor evolution, probably as a result of genome instability. Furthermore, all m-PTC cases without *EWSR1* monosomy had a high percentage of *EWSR1* rearrangement with unknown partner’s genes. Further studies for the identification of one or more partner genes are necessary and may lead to the identification of specific translocation related to the metastatic potential of m-PTC.

Considering the association of *EWSR1-FLI1* rearrangement with the presence of small-cell phenotype in mesenchymal neoplasm, this study analyzed the presence of small cells in *EWSR1-FLI1* positive m-PTC. The current study demonstrated a significant positive correlation between the presence of small cells and the percentage of cells with *EWSR1-FLI1* rearrangement in m-PTC. These findings were also supported by the results of the nuclei perimeter measurements, being the smallest size of m-PTC nuclei are the nuclei positive for *EWSR1-FLI1* fusion. This finding is in line with the idea that this translocation has a role in the acquisition of small-cell phenotype, not only in mesenchymal neoplasms but also in thyroid tumors, specifically CEFTE, and now, in m-PTC [13,14]. The small-cell phenotype can be present in a wide variety of secondary and rarely primary thyroid tumors, such as medullary thyroid carcinoma (MTC), poorly differentiated thyroid carcinoma (PDTC) and lymphoma [16,26]. In general, a designation of thyroid malignancy as small cell thyroid carcinomas is related to the aggressive clinical course of these tumors [27]. The small cell variant of MTC tends to be more aggressive than conventional MTC [16]. The situation is not so uniform in PDTC where the presence of cells with small size is associated with more aggressive behavior of oncocytic PDTC, at variance to its conventional variant when the occurrence of small cells does not influence prognosis [28].

In m-PTC, the most important diagnostic criterion is the presence of nuclear changes [16] regardless of the size of the cells. The presence of a small-cell phenotype in PTC was not documented in the literature until now, but in few studies about PTC morphology, the presence of cells with different appearances has been discussed. According to our methodology, at least in part, cells that we recognized as small, corresponding to previously described unusual cellular forms of PTC, such as cells with a dark nucleus [29], dormant cells [30] or cells with incomplete nuclear features of PTC [31,32].

The meaning of these unusual cellular forms previously described is unknown, but it was assumed that they have been sequestered from the normal cell cycle because they did not show proliferative activity [30]. In another study, it was reported that the presence of cells with dark nuclei were a more common finding in malignant thyroid tumors than in benign one, and their presence was assumed to be useful assisting the diagnosis of malignancy, specifically in difficult cases of follicular lesions suspected for a follicular variant of PTC [29].

In the present series, the association of small cells-positive m-PTC was observed between the presence of invasive growth, lymphatic invasion, and moderate/extended intratumoral fibrosis of m-PTC. These findings suggest a possible role of small cells in the aggressiveness of m-PTC like it was previously shown for lymphatics invasion and intratumoral fibrosis [22,33]. The development of intratumoral fibrosis required time, and since most m-PTC represents old lesions, an­ ­association of intratumoral fibrosis and small cells may indicate that acquisition of small-cell phenotype is a consequence of m-PTC aging. Future studies of small cells in the larger series of m-PTC with a long time follow and with a comparison of small cell significance in larger PTC, may be useful in determining their clinical significance.

In the group of mesenchymal neoplasms, the *EWSR1-FLI1* rearrangement has been related to an aggressive clinical course [3,4] at variance with what is documented in the *EWSR1-FLI1* positive m-PTC of the current series. This could be explained by the fact that *EWSR1-FLI1* positive cells represent only a small cellular clone in m-PTC which was not sufficient to induce the change of tumor behavior with a consequent aggressive course. However, *EWSR1-FLI1* rearrangement is present in all tumor cells of CEFTE and a small-cell phenotype is completely developed, but prognosis so far is favorable [13-15]. On the contrary, rare cases of pancreatic neuroendocrine tumors with *EWSR1-FLI1* rearrangement were not showing small-cell phenotype and other clinicopathological features of ESFT [34]. Accordingly, the acquisition of specific tumor phenotype does not depend only on the presence of *EWSR1-FLI1* fusion. The same fusion transcript could induce phenotype change only in the right cellular context because there are significant phenotype differences in various tumors that arise from the different cell types with identical gene fusion [3,4]. As well, additionally, genetic and epigenetic events that can also influence of *EWSR1-FLI1* protein activity are necessary as its continuous expression is required to maintain the oncogenic phenotype of ESFT cells, acquisition of specific tumor phenotype and clinical behavior [3,4,35,36].

## CONCLUSION

*EWSR1-FLI1* rearrangement is not rare in m-PTC and it is related to the acquisition of small-cell phenotype. The molecular alterations of the *EWSR1* gene, monosomy, and rearrangements, are frequent events in m-PTC, they have, apparently, no clinical significance and are related to the classic pattern of m-PTC.
